# Carotenoid Production by *Dunaliella salina* under Red Light

**DOI:** 10.3390/antiox8050123

**Published:** 2019-05-07

**Authors:** Yanan Xu, Patricia J. Harvey

**Affiliations:** Faculty of Engineering and Science, University of Greenwich, Central Avenue, Chatham Maritime, Kent ME4 4TB, UK; y.xu@greenwich.ac.uk

**Keywords:** *Dunaliella salina*, microalgae, red LED, blue LED, growth, carotenoids, plastoquinol:oxygen oxidoreductase, photosynthesis

## Abstract

The halotolerant photoautotrophic marine microalga *Dunaliella salina* is one of the richest sources of natural carotenoids. Here we investigated the effects of high intensity blue, red and white light from light emitting diodes (LED) on the production of carotenoids by strains of *D. salina* under nutrient sufficiency and strict temperature control favouring growth. Growth in high intensity red light was associated with carotenoid accumulation and a high rate of oxygen uptake. On transfer to blue light, a massive drop in carotenoid content was recorded along with very high rates of photo-oxidation. In high intensity blue light, growth was maintained at the same rate as in red or white light, but without carotenoid accumulation; transfer to red light stimulated a small increase in carotenoid content. The data support chlorophyll absorption of red light photons to reduce plastoquinone in photosystem II, coupled to phytoene desaturation by plastoquinol:oxygen oxidoreductase, with oxygen as electron acceptor. Partitioning of electrons between photosynthesis and carotenoid biosynthesis would depend on both red photon flux intensity and phytoene synthase upregulation by the red light photoreceptor, phytochrome. Red light control of carotenoid biosynthesis and accumulation reduces the rate of formation of reactive oxygen species (ROS) as well as increases the pool size of anti-oxidant.

## 1. Introduction

Carotenoids are orange, yellow or red pigments which are synthesized by all photosynthetic organisms for light-harvesting and for photo-protection, and for stabilising the pigment–protein light-harvesting complexes and photosynthetic reaction centres in the thylakoid membrane. They may also be accumulated by some non-photosynthetic archaea, bacteria, fungi and animals for pigmentation [1,2,3]. Carotenoids are also the precursors of a range apocarotenoids of biological and commercial importance, such as the phytohormone abscisic acid, the visual and signalling molecules retinal and retinoic acid, and the aromatic or volatile beta-ionone [4]. Increasingly sought after as natural colorants, there is accumulating evidence that carotenoids protect humans against ageing and diseases that are caused by harmful free radicals and may also reduce the risks of cataract, macular degeneration, neurodegeneration and some cancers [5,6]. They have also been implicated as the actives for treating diseases associated with retinoids [4].

In most plants and algae containing chlorophyll a (λ_max_ ~680 nm) and b (λ_max_ ~660 nm), photons with a wavelength of 660–680 nm yield the highest quantum efficiencies. However the solar spectrum at the surface of the Earth is at its maximum intensity in the blue and green regions of the visible spectrum (400–550 nm), which is where carotenoids have strong absorption. In photosynthetic organisms in the light, carotenoids drive photosynthesis by transferring absorbed excitation energy to chlorophylls, which have poor absorption in this range. Carotenoids are also able to protect photosynthetic organisms from the harmful effects of excess exposure to light by permitting triplet–triplet energy transfer from chlorophyll to carotenoid and by quenching reactive oxygen species (ROS) [2].

*Dunaliella salina*, a halotolerant chlorophyte, is one of the richest sources of natural carotenoids and, similar to various members of the Chlorophyceae, accumulates a high content (up to 10% of the dry biomass) of carotenoids under conditions that are sub-optimal for growth i.e., high light intensity, sub-optimal temperatures, nutrient limitation and high salt concentrations. In *D. salina*, the major accumulated carotenoid is β-carotene, which is stored in globules of lipid and proline-rich, carotene globule protein in the inter-thylakoid spaces of the chloroplast (βC-plastoglobuli) [7,8,9,10]. The pathway for β-carotene synthesis and accumulation in *D. salina* has been partly mapped out [11,12], but the physiological role and signals triggering its accumulation are not well established. In other members of the Chlorophyceae, such as *Haematococcus pluvialis* and *Chlorella zofingiensis*, high levels of oxygen-rich, secondary ketocarotenoids, astaxanthin and canthaxanthin, also accumulate under high light stress or nutrient stress, often in lipid bodies located outside the chloroplast in the cytoplasm. Accumulation of these may also be accompanied by cell encystment. Lemoine and Schoefs [13] proposed that these carotenoids accumulate as a metabolic means of lowering ROS levels by lowering cellular oxygen concentration, as well as serving as a convenient way to store energy and carbon for further synthesis under less stressful conditions [13,14]. Chemically generated ROS will trigger astaxanthin accumulation [15] and recently Sharma et al. [16] showed that a small dose (up to 50 mJ cm^2^) of short wavelength ultraviolet C (UV-C ) light (100–280 nm) in cultures of either *D. salina* or *H. pluvialis* massively increased carotenoids accumulation as well as detached the flagellae to increase cell settling, 24 h after exposure: UV-light exposure is typically accompanied by ROS formation.

However in *D. salina* there may be additional mechanisms leading to carotene accumulation. Jahnke [17] for example found that whilst supplements to visible radiation of long wavelength ultraviolet A (UV-A) radiation (320–400 nm) specifically increased carotenoid levels and the ratio of carotenoids to chlorophylls in the closely related *D. bardawil,* neither blue light nor medium wavelength ultraviolet B (UV-B) light (290–320 nm) supplements were similarly effective. In blue light, Loeblich [8] found that green cells of *D. salina* with a low carotenoid to chlorophyll ratio had a relatively depressed photosynthetic activity, which was even more exaggerated in red cells with a high carotenoid to chlorophyll ratio. They proposed that blue light, which was absorbed by the accumulated β-carotene, was not available for photosynthetic oxygen evolution. Amotz et al. [18] on the other hand found a marked photo-inhibition for both red and green cells under high intensity red light, which is absorbed by chlorophylls, but red cells, when transferred to high intensity blue light were seemingly photoprotected. Since the accumulated carotenoids were physically distant from chlorophylls located in thylakoid membranes, Amotz et al. [18] proposed that in high intensity red light, the carotenoids were unable to provide photoprotection against chlorophyll-generated ROS or quench chlorophyll excited states, supporting the argument that carotene globules may function as a screen against high irradiation in blue light to protect photosynthetic reaction centres in *D. salina*. Fu et al. [19] examined the effects of different light intensities of red LED light on carotenoid production in *D. salina,* and showed that the major carotenoids changed in parallel to the chlorophyll b content and that both carotenoids and chlorophyll b decreased with increasing red light intensity and increased with nitrogen starvation.

Light-emitting diodes (LEDs) can be applied to adjust the biochemical composition of the biomass produced by microalgae via single wavelengths at different light intensities [20,21,22,23] and recently Han et al. [20] successfully used a low light intensity blue-red LED wavelength-shifting system to increase carotenoid productivity in *D. salina*. In this paper we explore the effects of red, blue and white LEDs on the growth and content of carotenoids and chlorophyll in four different *D. salina* strains under nutrient-sufficient conditions using a temperature-controlled photobioreactor (PBR) favouring growth. We show that in this system, cultivation using red LED was particularly effective in supporting a high rate of carotenoid productivity. We suggest that in strains of *Dunaliella salina*, accumulating carotenoids may be synthesised principally as a mechanism for maintaining cellular homeostasis under conditions which might otherwise lead to over-reduction of electron transport chains, formation ROS and of a hyperoxidant state and ultimately lead to cell death.

## 2. Materials and Methods

### 2.1. Strains and Cultivation

Strains *D. salina rubeus* CCAP 19/41 and *D. salina salina* PLY DF17 were isolated from a salt pan in Eilat, Israel. *D. salina* CCAP 19/40 was isolated from a salt pond in Monzon, Spain. Strain UTEX 2538 (*D. salina bardawil*) was purchased from the Culture Collection of Algae and Protozoa (CCAP), Scotland, UK.

Algae were cultured in 500 mL Modified Johnsons Medium [24] containing 1.5 M NaCl and 10 mM NaHCO_3_ in Erlenmeyer flasks (Fisher Scientific, UK) in an ALGEM Environmental Modelling Labscale Photobioreactor (Algenuity, Bedfordshire, UK) at 25 ℃. The cultures were shaken for 10 min at 100 rpm every hour before taking samples to monitor cell growth. Cells were grown under 12/12 light/dark (L/D) with 200 μmol photons m^−2^ s^−1^ supplied by white LED light to exponential growth phase and then dark-adapted for 36 h. After dark adaption, cultures were exposed continuously to blue, red or white LED light at light intensities of 200, 500, or 1000 μmol photons m^−2^ s^−1^. Cultures acclimated to white, red or blue LED light for 24 h were used to monitor the changes in cellular carotenoids after further growth for 24 h in white, red or blue LED light. Cell density of the cultures was determined by counting the cell number of cultures using a haemocytometer after fixing with 2% formalin.

### 2.2. Pigment Analysis

The composition of pigments was analysed by High-Performance Liquid Chromatography with Diode-Array Detection (HPLC-DAD) (Agilent Technologies 1200 series, Agilent, Santa Clara, United States), using a YMC30 250 × 4.9 mm I.D S-5µ HPLC column (YMC, Europe GmbH) at 25 ℃ with an isocratic solvent system of 80% methanol: 20% methyl tert-butyl ether (MTBE) and flow rate of 1 mL min^−1^ at a pressure of 78 bar. Carotenoid standards of β-carotene, α-carotene, lutein, zeaxanthin and phytoene were obtained from Sigma-Aldrich Inc. (Merck KGaA, Darmstadt, Germany) and dissolved in methanol or acetone to generate standard curves and DAD scans analysed at wavelengths of 280 nm (phytoene), 355 nm (phytofluene), 450 nm (β-carotene, α-carotene, lutein and zeaxanthin), and 663 nm (chlorophylls). Pigments were extracted from the biomass of 15 mL samples of culture. Samples were harvested by centrifugation at 3000× *g* for 10 min and pigments extracted after sonication for 20 s with 10 mL MTBE–MeOH (20:80). Samples were clarified at the centrifuge then filtered (0.45 µm filter) into amber HPLC vials before analysis.

Total carotenoids and total chlorophyll in the cultures were measured using a Jenway 6715 UV/Vis spectrophotometer (Cole-Parmer, Staffordshire, UK). Pigments were extracted from the harvested algal biomass of 1 mL culture using 1 mL of 80% (*v*/*v*) acetone, then clarified at 10,000× *g* for 10 min. The content of total carotenoids was calculated from absorbance values at 480 nm according to Strickland & Parsons [25]. Chlorophyll a, b and total chlorophyll content was measured at 664 nm and 647 nm according to Porra et al. [26].

### 2.3. Oxygen Evolution and Dark Respiration

Samples of cultures exposed to white, red or blue LED light were collected and the rates of O_2_ evolution and dark respiration were measured as described by Brindley at al. [27] at 25 ℃ using a Clark-type electrode (Chlorolab 2, Hansatech Instruments Ltd, Norfolk, UK). O_2_ evolution/uptake was induced by white, red, or blue LED light supplied by the manufacturer at a light intensity of 1000 μmol m^−2^ s^−1^. After an initial period of 30 min of dark adaption of 1.5 mL of each culture, the rate of O_2_ evolution/uptake was measured for 20 min followed by dark respiration for 20 min. The average rate of photosynthesis was determined from the linear rate of oxygen evolution during 5–15 min of the light period. Dark respiration was determined by following the same procedure, except that the rate was calculated using the data from the last 15 min of the measurement. Air saturated water and nitrogen were used to calibrate the electrode.

## 3. Results

### 3.1. Cell Growth and Carotenoids Production in Acclimated Cultures

**Figure 1:**
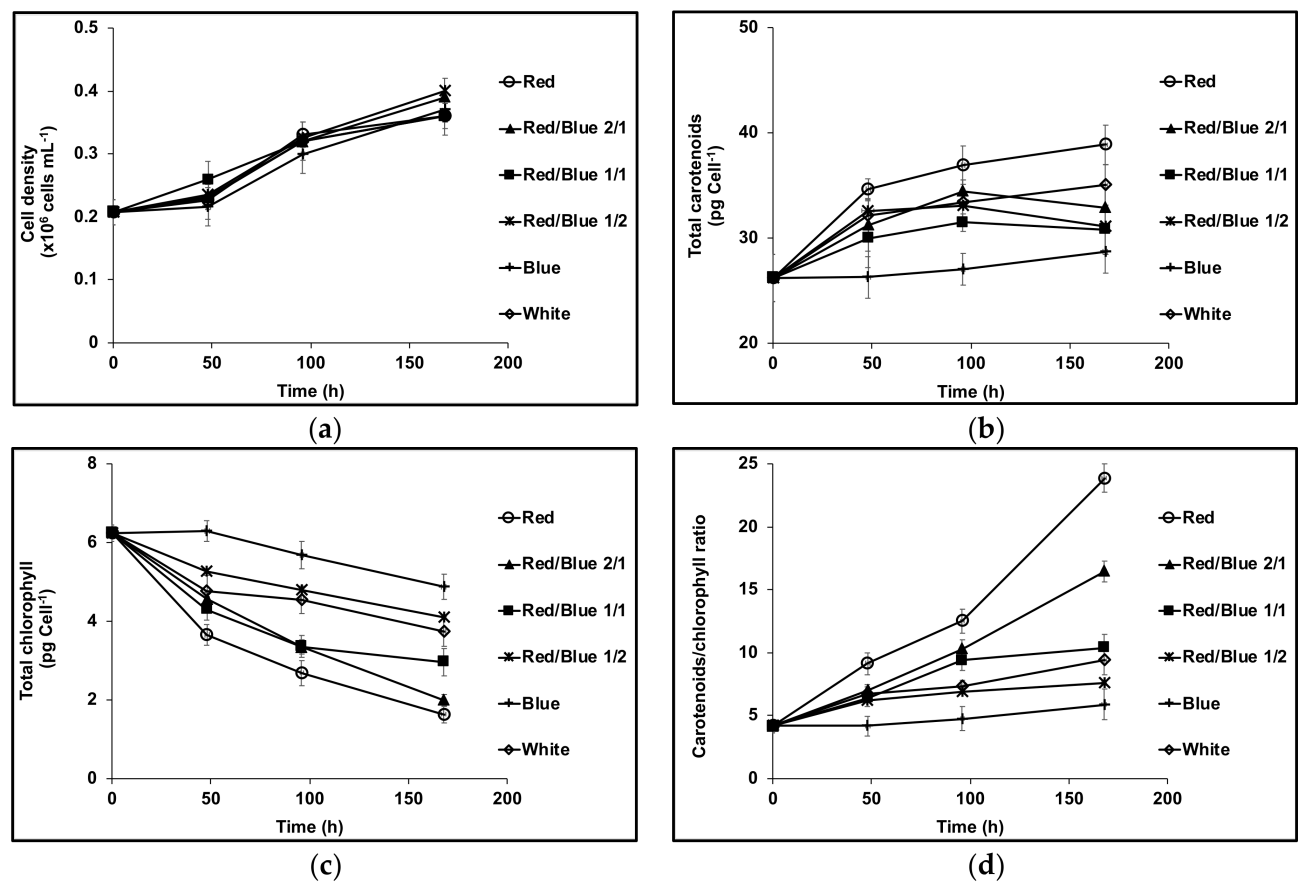
(**a**) Cell growth; (**b**) Cellular content of total carotenoids; (**c**) cellular content of total chlorophyll; (**d**) Carotenoids/Chlorophyll ratio in *D. salina* CCAP 19/41 grown under different ratios of red and blue light (Red/Blue 1/0, 2/1, 1/1, 1/2, 0/1) or white light with a total light intensity of 1000 μmol photons m^−2^ s^−1^ after dark-acclimation. Each culture condition was set up at least in triplicate.

Figure 1a shows that in high intensity blue, white or red LED light, the growth rate recorded as cell density for *D. salina* CCAP 19/41 was the same. There was no significant difference in cell size (data not shown). However the contents of total carotenoids and total chlorophyll depended on the relative proportions of blue or red light supplied. The initial phase of growth in all high intensity light conditions, apart from blue, caused an initial sharp drop in chlorophyll content; the drop was greatest in high intensity red LED light but decreased depending on the relative proportions of red: blue light supplied. On the other hand, cultures maintained in red LED accumulated carotenoids at the highest rate; in blue, the content declined depending again on the relative proportions of red and blue light supplied (Figure 1b,c). The carotenoids/chlorophyll ratio is often used to evaluate carotenogenesis in *D. salina*. As shown in Figure 1d, the ratio increased rapidly with the increasing proportion of red light supplied but remained the same for blue light.

**Figure 2:**
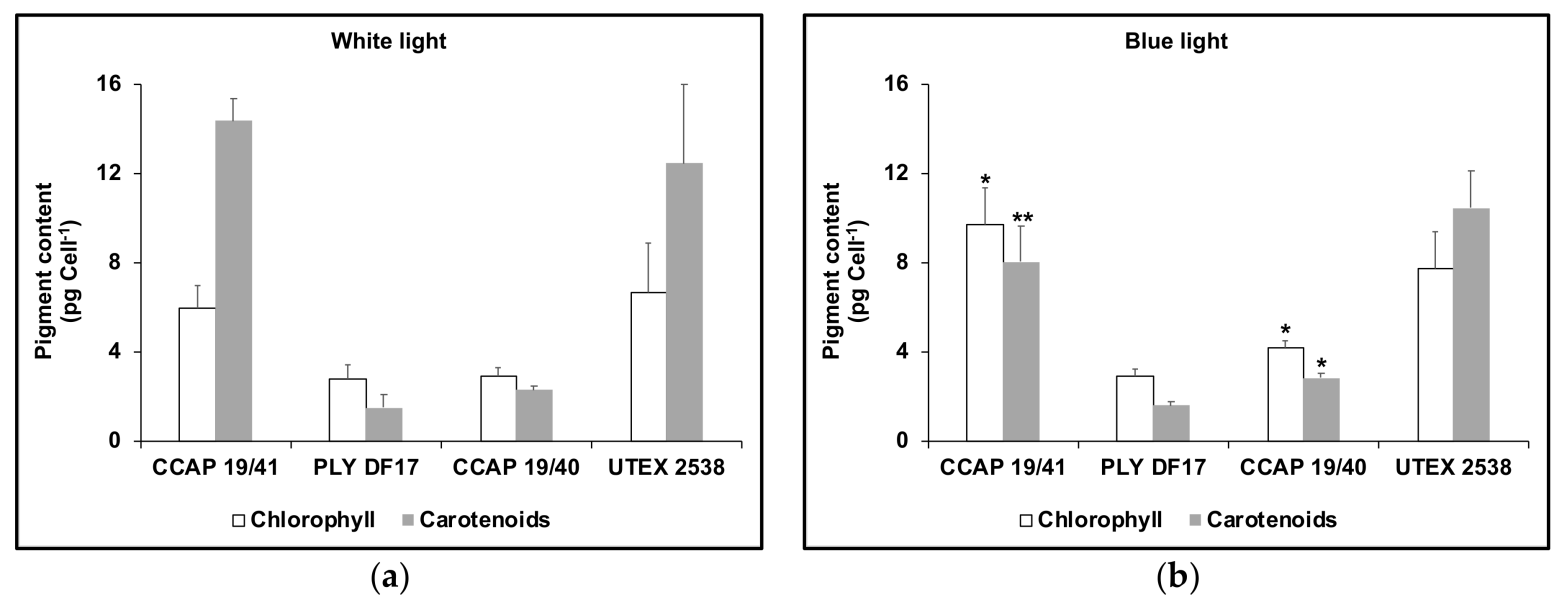
Cellular content of total carotenoids and of total chlorophyll for different *D. salina* strains cultivated each to the mid log phase under either white (**a**) or blue (**b**) light with a total light intensity of 1000 μmol photons m^−2^ s^−1^ after dark-acclimation. Each culture condition was set up at least in triplicate. Results were analysed by one way ANOVA comparing blue light to white light, **: 0.001 < *p* ≤ 0.01; *: 0.01 < *p* ≤ 0.05.

Different *Dunaliella* strains responded differently to cultivation in high intensity blue or white LED (see Figure 2). All showed a decline in chlorophyll content in white LED compared to cultivation in high intensity blue but only strain CCAP 19/41 showed a significant increase in carotenoid content in white compared to blue light.

**Figure 3:**
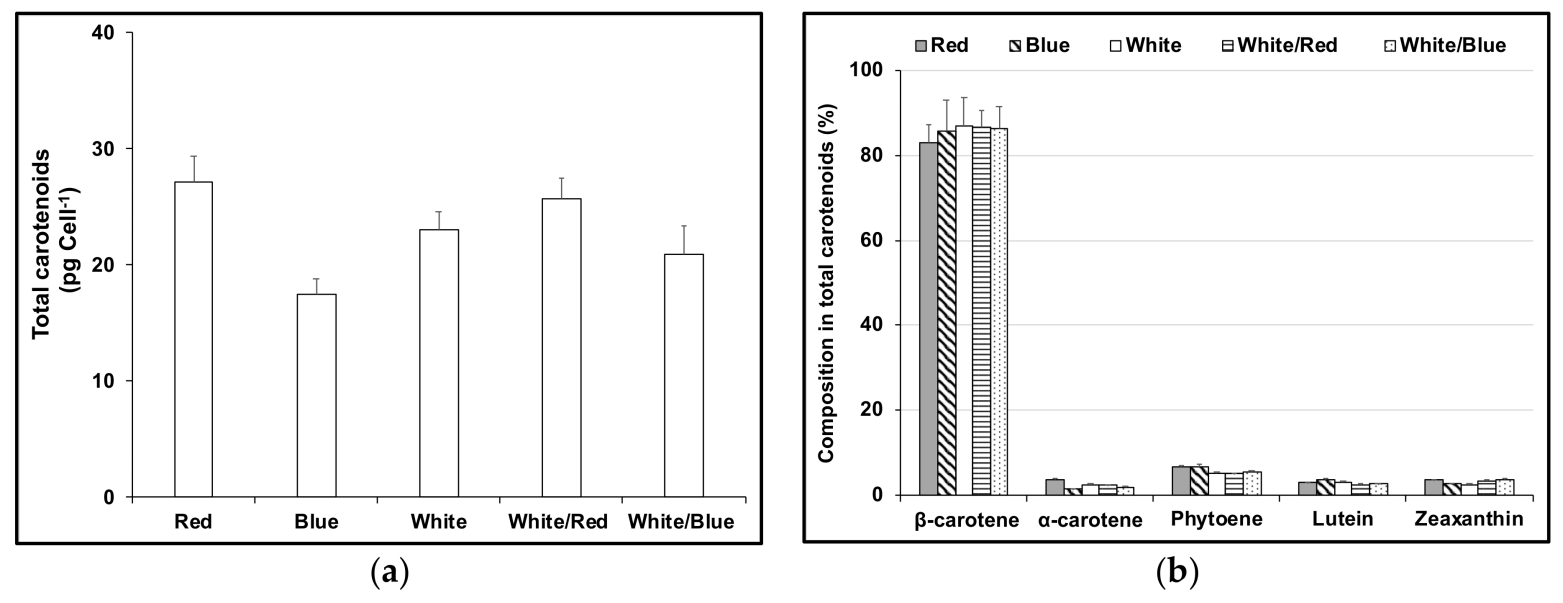
(**a**) Cellular content of total carotenoids; (**b**) Relative composition of major carotenoids characterised by HPLC in total carotenoids in *D. salina* CCAP19/41 cells exposed to continuous LED of different wavelength distribution (red, blue, white, white/red 1:1, and white/blue 1:1) for 48 h. The total light intensity for all conditions was the same at 1000 μmol photons m^−2^ s^−1^. Results were analysed by one way ANOVA.

Carotenoids in *D. salina* CCAP 19/41 cultures exposed to different light conditions: white, red or blue LED light at 1000 μmol photons m^−2^ s^−1^, a mixture of white and red (1:1) or a mixture of white and blue (1:1) with a total intensity of 1000 μmol photons m^−2^ s^−1^ for 48 h were extracted and the major carotenoids were identified and quantified by HPLC. Cultures exposed to continuous red LED light had the highest contents of all the identified carotenoids, while cultures maintained under blue LED light showed the lowest content. The difference was mainly due to variation in β-carotene content between treatments: there was no significant difference in relative content of all other carotenoids (Figure 3).

**Figure 4:**
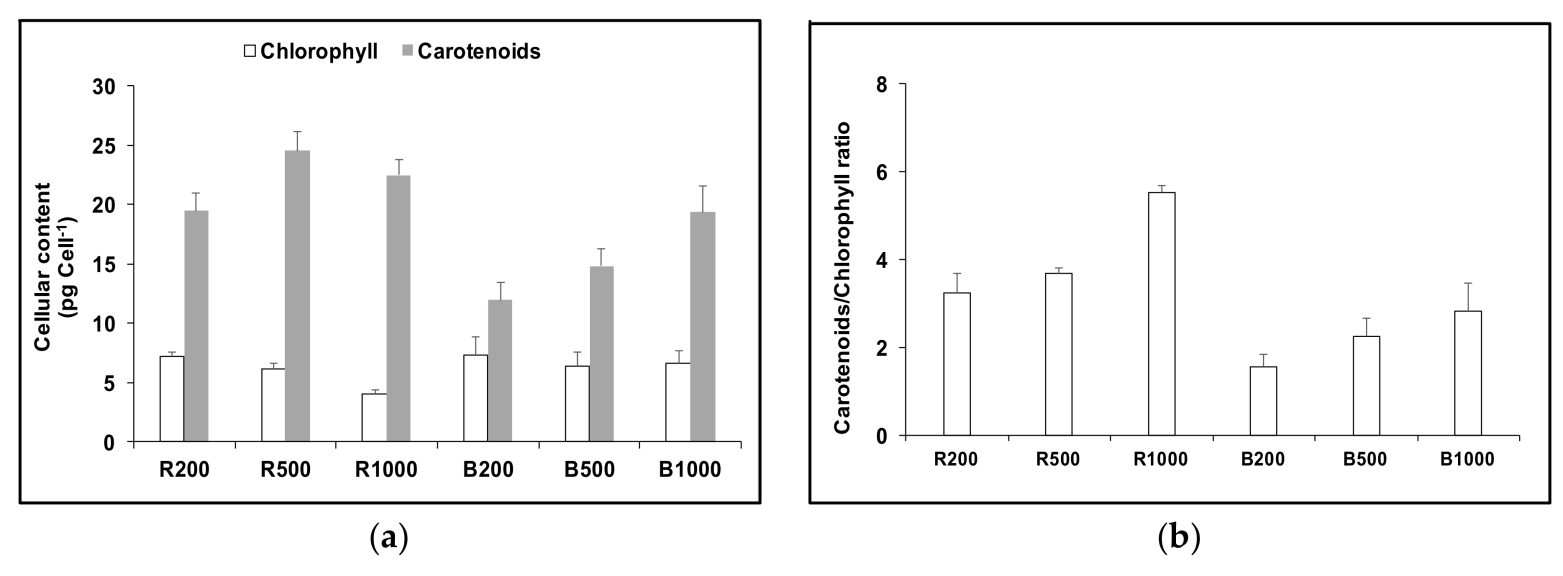
Cellular content of total carotenoids and total chlorophyll (**a**) and carotenoids/chlorophyll ratio (**b**) in *D. salina* CCAP19/41 grown under continuous red (R200; R500; R1000) or blue (B200; B500; B1000) LED light at three different light intensities of 200, 500 and 1000 µmol m^−2^ s^−1^ for 48 h. Each culture condition was set up at least in triplicate.

The total carotenoids and total chlorophyll contents after 48 h exposure to red or blue LED at different light intensities are shown in Figure 4. Carotenoids accumulated with increasing blue LED intensity between 200 μmol m^−2^ s^−1^ and 1000 μmol m^−2^ s^−1^. In red LED light, cultures contained high amounts of carotenoids even under low light intensity and the content increased with increasing red LED intensity up to 500 μmol m^−2^ s^−1^. With further increase in light intensity to 1000 μmol m^−2^ s^−1^, carotenoids declined slightly (<10% of the value recorded at 500 μmol m^−2^ s^−1^), but chlorophylls declined 34%. The carotenoids/chlorophyll ratio increased with the increase of light intensity both red and blue LED light, however, under red LED light a much higher carotenoids/chlorophyll ratio was recorded than under blue. Cellular content of β-carotene and phytoene showed a similar trend to that of total carotenoids, except that the highest β-carotene content under red light was achieved at 500 μmol m^−2^ s^−1^, while the highest phytoene content under red light was achieved at 1000 μmol m^−2^ s^−1^.

**Figure 5:**
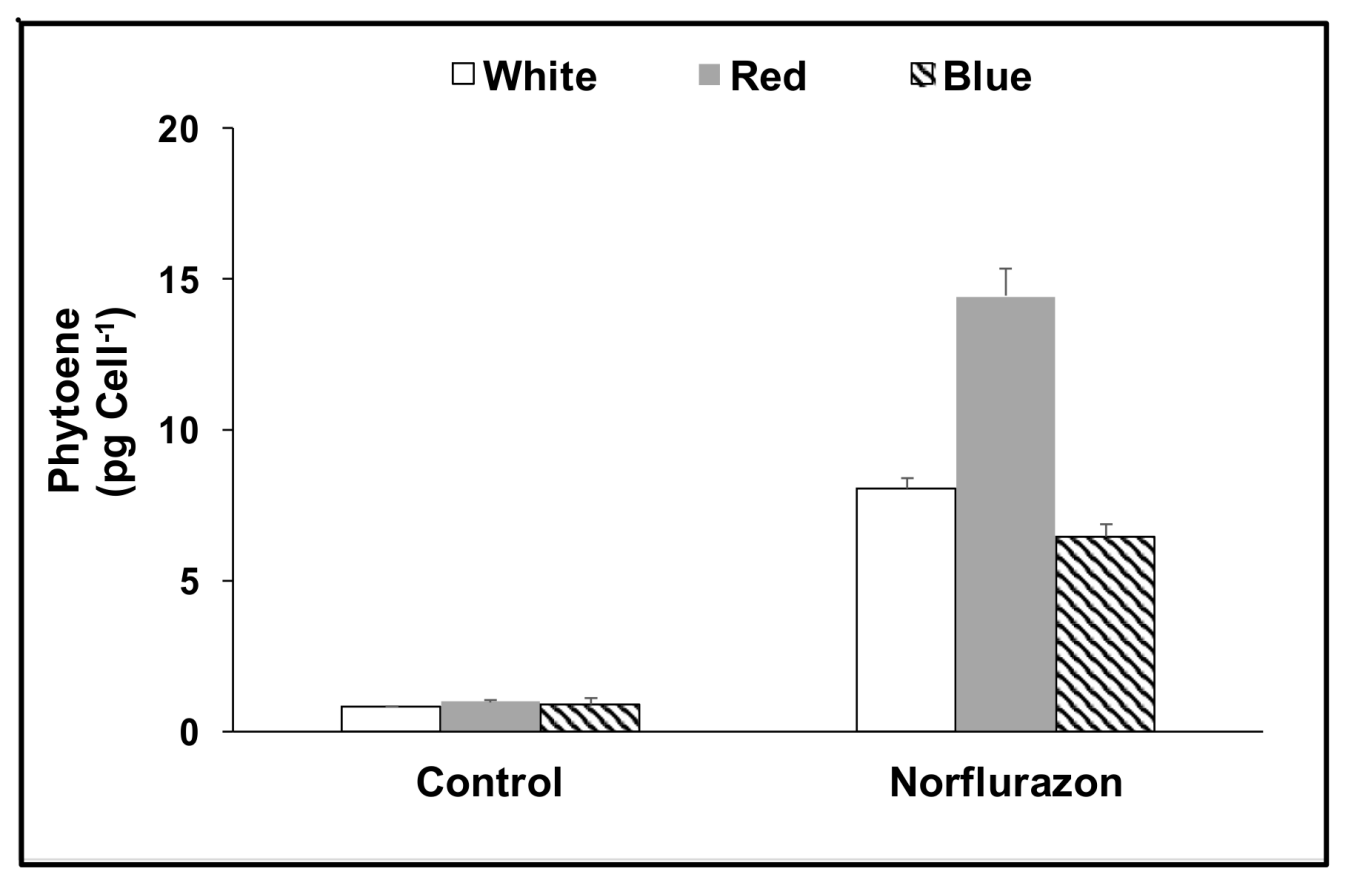
Cellular content of phytoene in cultures treated with no inhibitors (control) or with 5 µM norflurazon. Cultures were maintained under red, blue and white LED light at 200 µmol m^−2^ s^−1^ for 48 h.

A phytoene desaturase inhibitor norflurazon known to cause accumulation of phytoene was used to treat *D. salina* cultures maintained under red, blue or white LED light. Figure 5 shows that under these conditions the cellular content of phytoene increased, as expected, but cultures maintained under red LED accumulated a significantly higher amount of phytoene compared to cultures maintained under white or blue light.

### 3.2. Acclimation and Carotenoids Production in Response to Wavelength Switching

**Figure 6:**
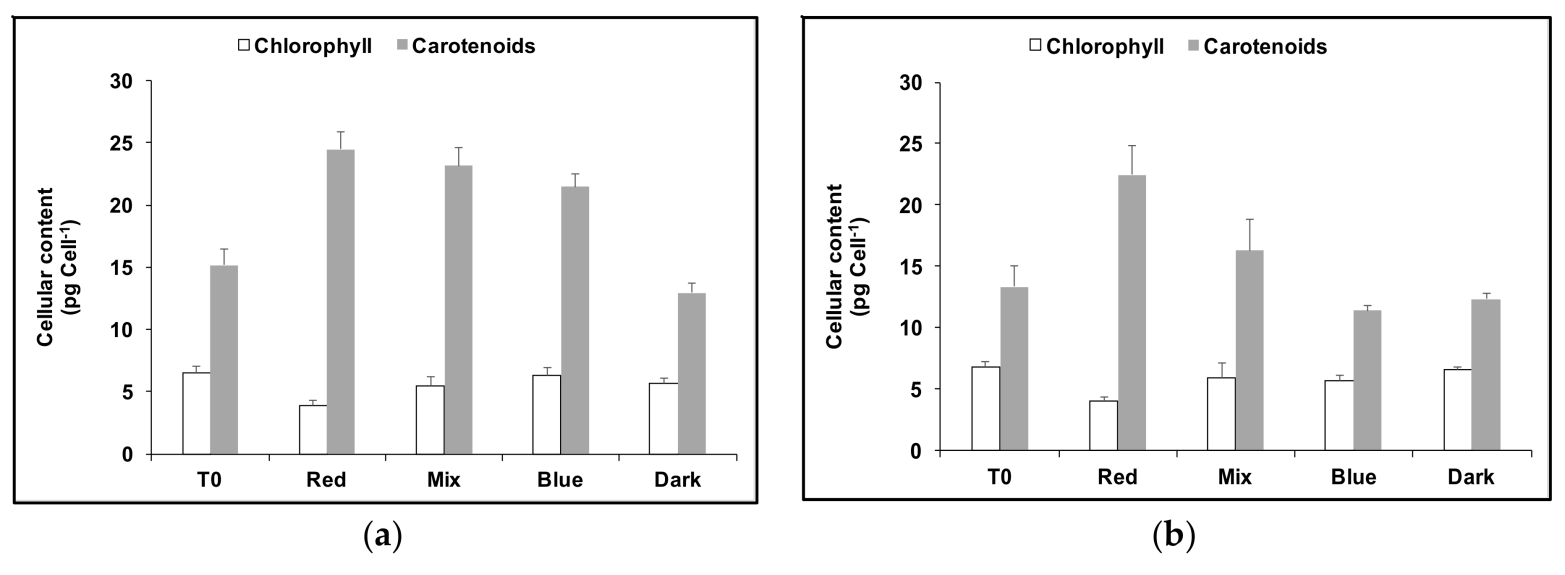
Cellular content of total carotenoids and chlorophyll under continuous blue (**a**) or red (**b**) LED light at 1000 µmol m^−2^ s^−1^ for 24 h followed by 24 h growth under either red light, a mix of 1:1 red and blue light, blue light at the same light intensity of 1000 µmol m^−2^ s^−1^ or dark. Each culture condition was set up at least in triplicate. T0: time point after growth for 24h only, in either blue (a) or red (b) light at 1000 µmol m^−2^ s^−1^.

Dark-adapted cultures of *D. salina* CCAP19/41 were cultivated in red, white or blue LED light for 24 h (T0), and then cultivated for a further 24 h in red, blue, or a mixture of red and blue LED light (1:1), or the dark. Blue-acclimated cells produced slightly more carotenoids (14% greater content) when transferred to red LED but chlorophyll content declined from that at the start of the experiment to an amount only 62% of that in continuous blue (Figure 6a). On the other hand the chlorophyll content increased when red-acclimated cells were exposed to blue light, but the total carotenoids content declined sharply, approximately in proportion to the amount of blue LED supplied (see Figure 6b). Red LED cultures maintained for a further 24 h in red LED accumulated 24.5 ± 1.3 pg carotenoid cell^−1^, but after 24 h in blue LED instead of red, the carotenoid content was 50% lower (11.4 ± 0.4 pg carotenoid cell^−1^) and less than if they had been transferred to the dark (12.3 ± 0.5 pg carotenoid cell^−1^).

**Figure 7:**
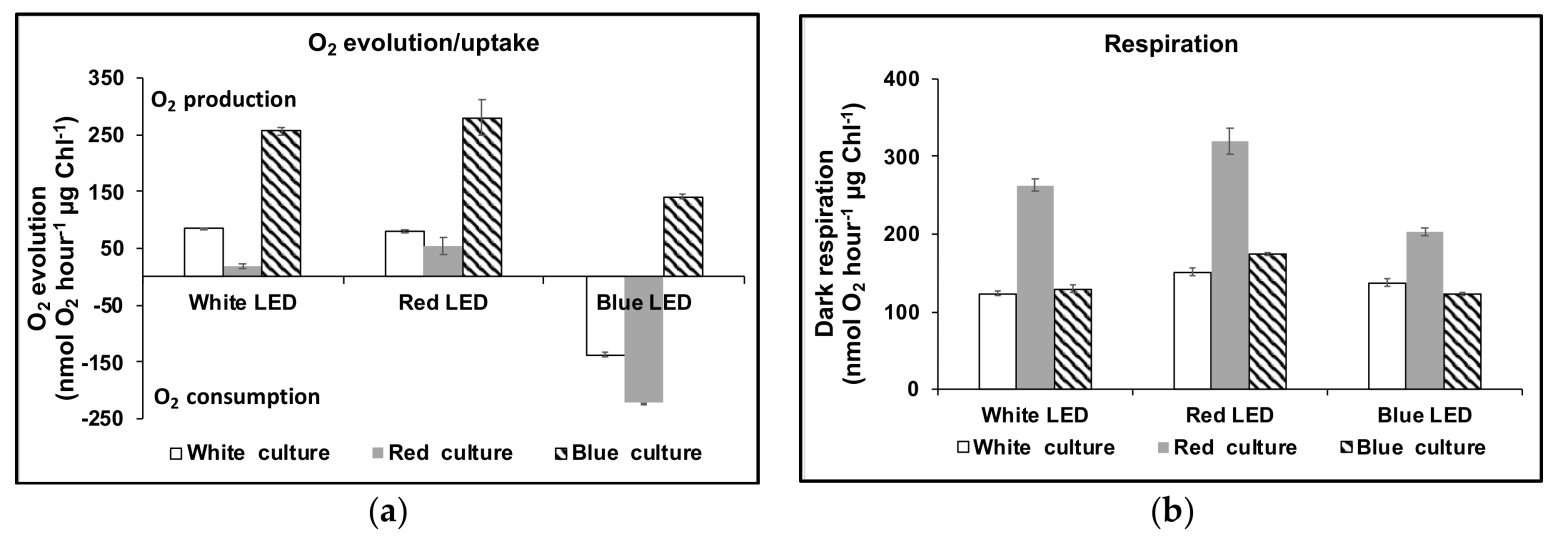
Oxygen evolution/uptake by *D. salina* cultures in different white, red or blue LED light sources (**a**) and in the dark (**b**). Cultures were grown under continuous red, blue or white light at 1000 µmol m^−2^ s^−1^ for 48 h before measurement.

**Figure 8:**
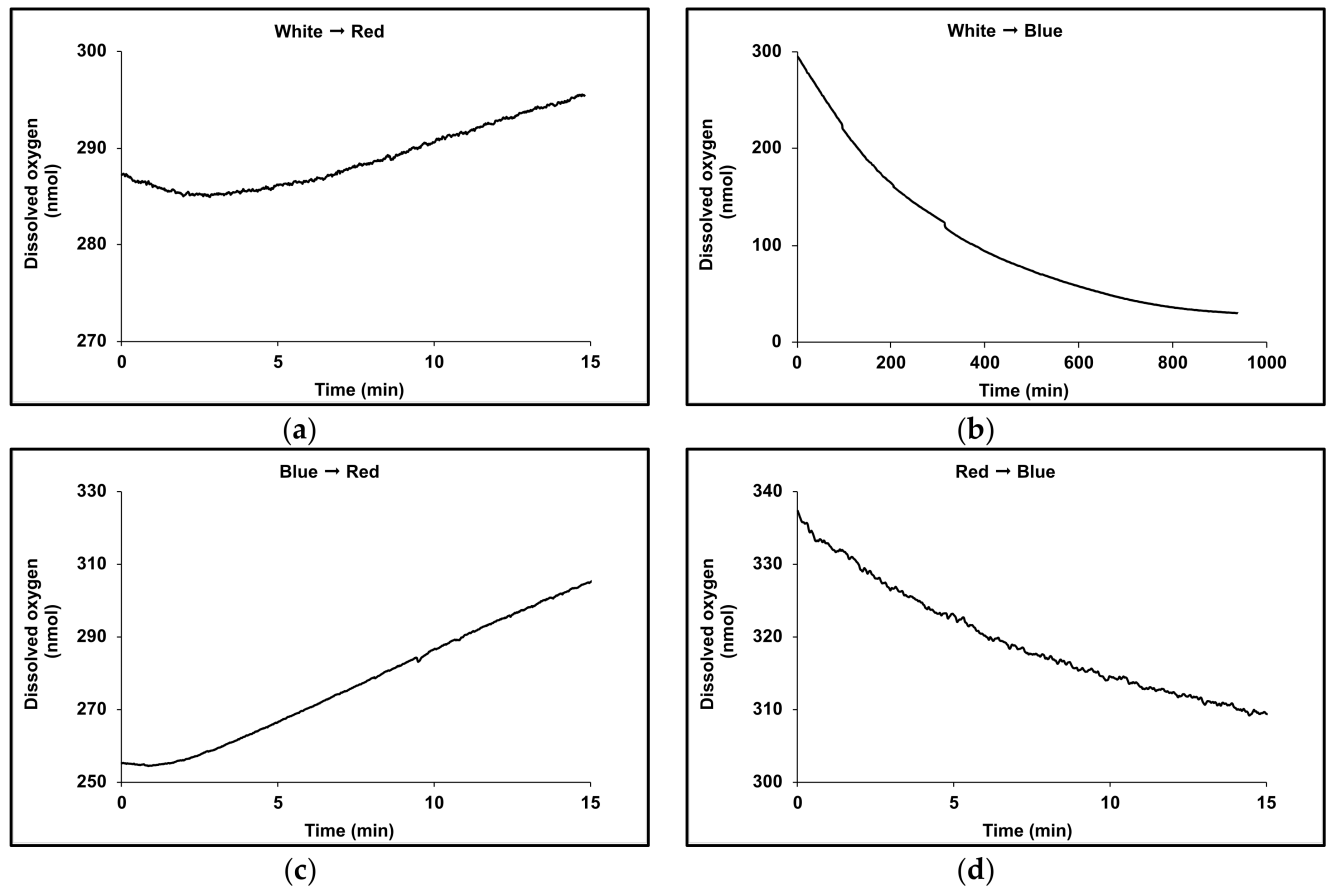
Rate profiles for oxygen uptake/evolution measured with different wavelengths of LED lights measured using a Clark-type electrode for 1.5 mL cultures of *D. salina* CCAP19/41 maintained at 25 ℃. (**a**) Red light acclimated cultures measured under blue light. (**b**) Blue light acclimated cultures measured under red light; (**c**) White light acclimated cultures measured under red light; (**d**) White light acclimated cultures measured under blue light.

Dark-adapted cultures were cultivated in red, blue or white light at 1000 µmol m^−2^ s^−1^ for 48 h before measuring the rates of oxygen evolution/uptake over a 20 min period with illumination supplied once more by white, red or blue LED lights at 1000 µmol m^−2^ s^−1^ (Figure 7a). Dark respiration was also recorded (Figure 7b). The rate profiles of oxygen uptake/evolution are shown in Figure 8. Red LED supported net oxygen evolution (55 ± 15 nmol O_2_ h^−1^ μg chlorophyll^−1^) but on transfer to blue light in the Clark-type electrode, photo-oxidation massively exceeded the rate of oxygen evolution and oxygen was consumed at an exponentially increasing rate (Figure 7a; 222 ± 32 nmol O_2_ h^−1^ μg chlorophyll^−1^). Significantly cultures grown in red LED also supported the highest rate of dark respiration (320 ± 17 nmol O_2_ h^−1^ μg chlorophyll^−1^, ~2.6-fold greater than that for cultures maintained in either blue or white LED light), but this also declined when cultures were transferred to blue light in the Clark-type electrode. By contrast, cultures maintained in blue LED supported ~3-fold higher rate of net oxygen evolution (141 nmol O_2_ h^−1^ μg chlorophyll^−1^) in the Clark-type electrode in blue light, compared to those in maintained in red LED. On transfer of blue light cultures to red light in the Clark-type electrode, the rate of oxygen evolution doubled to 280 nmol O_2_ h^-1^ μg chlorophyll^−1^ (Figure 7b), and was maintained at a linear rate during the period of measurement. The rate of dark respiration also increased slightly from 123 ± 2.7 nmol O_2_ h^−1^ μg chlorophyll^−1^ to 175 ± 5.0 nmol O_2_ h^−1^ μg chlorophyll^−1^.

## 4. Discussion

LEDs with different wavelengths have been increasingly used to study the wavelength effects on the growth and productivity of photoautotrophic microalgae, and much effort is being invested to understand the most energy-efficient way to incorporate their use for large-scale algal cultivation [20,21,22,23,28,29]. In the present work we explored the effects of using red, white, blue and mixtures of red and blue LEDs at different intensities to evaluate the basis for carotenoid accumulation in strains of *Dunaliella salina*.

The emission spectrum of the red LED used in the present work (625–680 nm) emits photons with the exact range required by molecules of chlorophyll a and chlorophyll b to initiate photosynthesis [30]. In *D. salina,* action spectra of O_2_ evolution rates show maximum photosynthetic activity within the red absorption bands of the chlorophylls [8]. Photosystems I and II (PSI, PSII), which both contain chlorophyll a, work together in a series of more than 40 steps that proceed with the efficiency of nearly 100% to transfer electrons from water to nicotinamide adenine dinucleotide phosphate in its oxidised form (NADP^+^) [2]. Consequently the wavelength range of the red LED should be the most efficient emission required for photosynthesis in this alga and deliver the highest specific growth rate. However this also depends on the rate at which the absorbed light energy from any given applied photon flux density is converted to chemical energy: with increasing photon flux density, photosynthesis eventually achieves a light-saturated maximum rate that is limited by the rate of carbon fixation in the Calvin cycle. *Spirulina platensis* for example exhibited the highest specific growth rate using high intensity red LED [28]. *C. reinhardtii* however, showed unstable growth in high intensity orange-red and deep red LED, which ceased completely after a few days and was accompanied by cell agglomeration [22]: agglomeration is typical of oxidant stress and formation of a hyperoxidant state [31].

In the present work, we found that in high intensity red light in conditions of nutrient sufficiency, *D. salina* strains maintained a growth rate at least equal to that in white light or blue LED light, seemingly in contrast with the work of others [8,18,19]. However we also found that some but not all strains accumulated carotenoids rapidly, within 48 h of exposure. Carotenoids are known antioxidants that are synthesized by many microalgae as part of the battery of photoprotective mechanisms necessary to prevent photo-inhibition caused by photo-oxidation of photosynthetic reaction centres [2,32,33]. Photo-oxidation may occur in photon flux density levels that result in absorption of more light than is required to saturate photosynthesis. At the molecular level, when a photon is absorbed by a chlorophyll molecule, it enters a short-lived singlet excited state (^1^Chl*): the longer the excitation of ^1^Chl* lasts, which increases under saturating light conditions, the greater the chance that the molecule will enter the triplet excited state (^3^Chl*) via intersystem crossing. ^3^Chl* has a longer excitation lifetime and can transfer energy to the ground state of O_2_ to form singlet oxygen, ^1^O_2_, predominantly at the reaction centre of PSII and, to a lesser extent, in the light-harvesting complexes. Photo-oxidative damage occurs to the photosynthetic apparatus when species such as ^1^O_2_ react with fatty acids form lipid peroxides, setting up a chain of oxygen activation events that may eventually lead to a hyperoxidant state and cell death. Carotenoids may protect the photosystems by reacting with lipid peroxidation products to terminate these chain reactions; by scavenging ^1^O_2_ and dissipating the energy as heat; by reacting with ^3^Chl* to prevent formation of ^1^O_2_ or by dissipation of excess excitation energy through the xanthophyll cycle. It is tempting therefore to suppose that the differences observed by different workers simply reflects differences in carotenoids content between different strains, but this does not explain what triggered the differences in carotenoids content.

In the *D. salina* strain CCAP 19/41, accumulation of carotenoids was accompanied by the highest rate of O_2_ consumption and a low rate of net O_2_ evolution, which might imply ^1^O_2_ formation and ROS accumulation. In the non-photosynthetic, astaxanthin-accumulating yeast, *Phaffia rhodozyma*, artificially generated ^1^O_2_ was proposed to degraded astaxanthin to relieve feedback inhibition of carotenoid biosynthesis and also to induce carotenoid synthesis by gene activation [34]. However these authors also found that carotenoid biosynthesis was linked to O_2_ consumption by a cyanide-insensitive alternative oxidase, serving to consume oxygen without chemiosmotic synthesis of adenosine triphosphate (ATP). In *C. reinhardtii,* a specific thylakoid-associated, terminal plastoquinol:oxygen oxidoreductase has been identified with homology to the mitochondrial alternative oxidase [35]. The smaller rate of oxygen uptake compared to mitochondrial respiration suggested a function in directly coupling oxygen uptake and the exergonic reaction of plastoquinol oxidation with plastoquinone reduction by a phytoene/phytoene desaturase couple, to permit endergonic carotene desaturation without ATP involvement [35]. In *D. bardawil*, a decrease in oxygen consumption rate coupled to phytoene accumulation caused by norflurazon inhibition of phytoene desaturase also suggests a connection between direct desaturation of phytoene and chloroplastic oxygen dissipation [36].

**Scheme 1:**
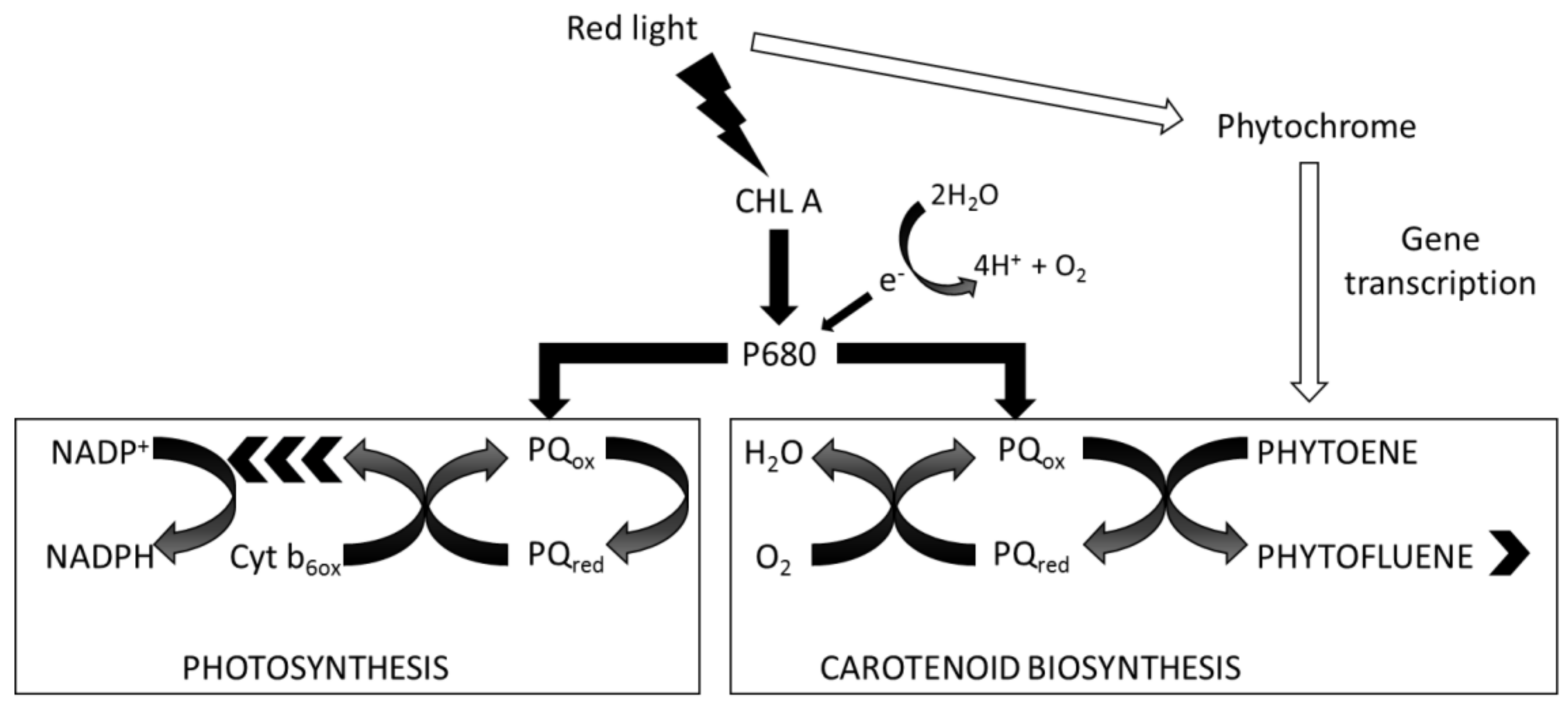
Partitioning electron flux between photosynthesis and carotenoid biosynthesis. Red photon flux intensity controls the partitioning of electrons either for carotenoid biosynthesis or for photosynthesis, via energy absorption by chlorophyll and the plastoquinone (PQ) pool. Red photon flux density also controls phytochrome regulation of phytoene synthase. CHL A: chlorophyll a; P680: chlorophyll a, primary electron donor of Photosystem II; PQ_ox_: plastoquinone, oxidised form; PQ_red_: plastoquinone, reduced form; Cyt b_6ox_: cytochrome b6f complex, oxidised form; NADP^+^: NADP oxidised form; NADPH: NADP reduced form.

In the present work, high intensity red light in conditions of nutrient sufficiency maintained growth at the same rate as in blue or white light, and red light also led to carotenoid accumulation albeit to different extents in different strains. These data support involvement of a plastoquinol:oxygen oxidoreductase as originally proposed for *C. reinhardtii* [35], but controlled by red photon flux intensity (see Scheme 1). In this scheme, chlorophyll absorption of red light photons is coupled to plastoquinone reduction in photosystem II, and oxygen reduction is coupled to phytoene desaturation by plastoquinol:oxygen oxidoreductase leading to carotenoid accumulation. Partitioning of electrons between photosynthesis and carotenoid biosynthesis would depend on both red photon flux intensity as well as upregulation of phytoene synthase. The observed increase in O_2_ consumption coupled to accumulation of carotenoids via the carotenoid biosynthetic pathway would reduce the tendency for ^1^O_2_ formation under high photon flux and maintain cytosolic redox potential.

The coupling of reduction of the plastoquinone pool to carotenoid synthesis driven by chlorophyll absorption of red light may involve the red light photoreceptor phytochrome. Photosynthetic organisms are known to perceive red light signals via phytochrome. The synthesis of phytoene by phytoene synthase is under phytochrome regulation [37,38,39] and is upregulated by both red and far-red light [37]. Red light also lowers the concentration of the transcription factor PIF1, a repressor of carotenoid biosynthesis [40]. In those strains which do not accumulate carotenoids, alternative mechanisms may serve to consume energy e.g., via NAD(P)H reduction of dihydroxyacetone phosphate to form glycerol [41].

In support of this model, transfer from high intensity red light to blue with higher energy content caused a massive drop in the accumulated carotenoid content, very high rates of photo-oxidation and low respiratory rates. Carotenoids in both the accumulated pool and in the light harvesting antenna, but not chlorophyll, absorb photons in the range 400–550 nm, exactly overlapping the emission spectrum of the blue LED (440–500 nm). Failure of chlorophyll molecules to use the absorbed energy to reduce the plastoquinone pool would be expected to reduce the rate of electron flux through the plastoquinol:oxygen oxidoreductase as well as uncouple carotenoid synthesis and consequently increase cellular O_2_ concentration. This would lead in turn to increased ROS formation by reaction of O_2_ with reduced electron transport chains, initiating further oxygen radical chain reactions, and carotenoid oxidation. Furthermore, in continuous high intensity blue LED, growth was maintained without carotenoid accumulation, but transfer to high intensity red LED light stimulated a small increase in carotenoid content, once again putting red light absorption by chlorophylls and transfer of absorbed energy to the plastoquinone pool at the centre of carotenoid biosynthesis. Transfer from blue to red light in the Clark-type electrode would cause absorption of more light than was required to saturate photosynthesis; if upregulation of the carotenoid biosynthetic pathway via phytochrome perception was required before coupling with O_2_ uptake via the plastoquinol:oxygen oxidoreductase, this would result in initial increase in the rate of photoinhibition and O_2_ uptake in the Clark-type electrode, and consequent loss in chlorophyll content, as was observed.

β-carotene accumulation in βC-plastoglobuli has parallels with that for astaxanthin accumulation, serving both as a carbon sink and end-product of an alternative oxygen-consuming biosynthetic pathway that on the one hand, controls over-reduction of photosynthetic (and respiratory) electron transport chains at the same time as removes oxygen from the plastid to limit formation of ROS. It is also able to quench any ROS that form. In blue light it may serve as a screen to absorb excess irradiation [7,18] but clearly offers photoprotection in red light as well. These functions are seen as distinct from its role as an accessory pigment in light-harvesting antennae systems.

Recently Davidi et al. [10] showed that the formation of cytoplasmic triacylglyceride (TAG) under N deprivation preceded that of βC-plastoglobuli, reaching a maximum after 48h of N deprivation and then decreasing. They suggested that βC-plastoglobuli are made in part from hydrolysis of chloroplast membrane lipids and in part by a continual transfer of TAG or of fatty acids derived from cytoplasmic lipid droplets. TAG synthesis represents a pathway for restricting over-reduction of electron transport chains [42] and its recruitment in formation of βC-plastoglobuli is entirely consistent with steps to dissipate excessive energy absorbed by chlorophyll in high intensity red light.

Overall, cultivation with red light may hold potential to enhance carotenoids production in carotenoid-accumulating strains of *D. salina*. Red light treatment has also been reported as an effective way to accelerate ripening of tomato fruit and increase the content of carotenoids [43]. Compared to other commonly used approaches to induce carotenogenesis, such as high light stress, high salt stress and addition of hydrogen peroxide or sodium hypochlorite, the use of red light provides a clean, convenient and economic alternative to promote carotenoids production from *D. salina* in a short time.

## 5. Conclusions

This study shows that under conditions of nutrient sufficiency, high intensity red light enhanced the production of carotenoids, mostly β-carotene, by upregulating the entire biosynthetic pathway of carotenoids, and that accumulation of carotenoids was accompanied by the highest rate of O_2_ consumption and a low rate of net O_2_ evolution. The data support a model of flexible co-operation between photosynthesis and carotenoid production via the plastoquinone pool. Chlorophyll absorption of red light photons and plastoquinone reduction in photosystem II is coupled to oxygen reduction and phytoene desaturation by plastoquinol:oxygen oxidoreductase. Partitioning of electrons between photosynthesis and carotenoid biosynthesis depends on photon flux intensity as well as upregulation of phytoene synthase by the red light photoreceptor phytochrome. Red light control of carotenoid biosynthesis and accumulation reduces the rate of formation of ROS as well as increases the pool size of anti-oxidant.

Red light may have industrial value as an energy-efficient light source for carotenoid production by *D. salina*.
